# Implementation and student outcomes of an implementation strategy for the sustainability of group evidence-based practices in schools

**DOI:** 10.21203/rs.3.rs-10164991/v1

**Published:** 2026-07-07

**Authors:** Ricardo Eiraldi, Rachel Comly, Tara Wilson, Muniya S. Khanna, Courtney Benjamin Wolk, Alexandra Bennett, Kathryn Henson, Kristina M. Popkin, Nicola Brooks, Patrick Bevenour, Jessica Goldstein, Abbas F. Jawad

**Affiliations:** 1 Children’s Hospital of Philadelphia, Roberts Center for Pediatric Research, 2716 South Street, Room 8293, Philadelphia, PA 19146-2305, USA;; 2 Department of Pediatrics, University of Pennsylvania Perelman School of Medicine, 3400 Civic Center Boulevard, Philadelphia, PA 19104, USA;; 3 Department of Psychiatry, University of Pennsylvania Perelman School of Medicine, 3535 Market St., Philadelphia, PA, 19104, USA;; 4 OCD and Anxiety Institute, 3138 Butler Pike # 200, Plymouth Meeting, PA, 19462, USA;; 5 Leonard Davis Institute of Health Economics, University of Pennsylvania, 3641 Locus Walk # 210, Philadelphia, PA, 19104, USA;; 6 School District of Philadelphia, 440 North Broad St., Philadelphia, PA 19130, USA

**Keywords:** Sustainability, Implementation, Effectiveness, Evidence-Based Practices, Urban schools

## Abstract

**Background:**

Limited research has examined the effectiveness and cost-effectiveness of training strategies to sustain mental health evidence-based practices (EBPs) in urban elementary schools.

**Methods:**

We conducted a hybrid type 2 cluster-randomized trial comparing Prepare for Sustainment (PFS)—school district coach supervision of implementers supported by external consultants—to Sustainment as Usual (SAU), which relied on coach supervision alone. Two EBPs were implemented: the Coping Power Program (CPP) for externalizing problems and CBT for Anxiety Treatment in Schools (CATS) for anxiety. The study included three phases: full support (Phase 1), reduced support (Phase 2), and no support (Phase 3). Participants included 40 master’s-level implementers, two district coaches, and 435 K–8 students. Outcomes included content and process fidelity, service penetration, mental health symptoms (parent and student report), and academic engagement with learning (teacher report). We also examined implementers’ perceptions of training.

**Results:**

PFS implementers demonstrated stronger process fidelity: greater engagement with students in Phase 2 and warmer, clearer, and more organized treatment delivery in Phase 3. Content fidelity and service penetration did not differ between conditions in either phase. PFS showed medium and small effect-size improvement on student behavioral and emotional engagement with learning in Phase 2, and a large effect size in behavioral engagement and a medium effect-size improvement in behavioral disaffection with learning in Phase 3 compared to SAU. Students receiving CPP in Phase 2 in the PFS condition demonstrated a large improvement on self-reported externalizing behavior, and students receiving CATS in Phase 2 showed a medium-size reduction in internalizing symptoms relative to students in SAU. Qualitative data showed that implementers who did not receive support from coaches desired supervision on effective EBP delivery, and those experiencing reduced support wanted more frequent guidance. Administrators noted that ongoing collaboration with district coaches would have enhanced their ability to support implementers.

**Conclusion:**

A sustainment strategy consisting of an initial training for implementers and occasional consultation for school district coaches is effective for achieving high process fidelity and medium to large effects for engagement with learning activities and mental health outcomes compared to a sustainment as usual strategy.

**Trial Registration:**

ClinicalTrials.gov identifier NCT04869657. Registered May 3, 2021

## Background

School-based mental health evidence-based practices (EBPs) are clinically effective and well-accepted by students and parents [1–3]. Despite their benefits, schools often struggle to sustain these practices over time [4–7]. A key barrier is limited professional training for implementers, such as school counselors [8, 9]. Research shows that initial training followed by ongoing supervision or consultation—especially when incorporating performance feedback—helps maintain implementers’ skills [10, 11]. Training is most effective when it reflects principles of adult learning and includes active learning strategies such as behavioral rehearsal [12, 13]. Consistent with this evidence, ongoing supervision or consultation have been identified as essential for sustaining EBPs in schools [14, 15], and reductions in post-training support are associated with practice abandonment [16]. However, sustaining full supervision or consultation is often not feasible in under-resourced school settings [17], highlighting the need for more efficient training models that preserve outcomes while minimizing burden.

This study compared a targeted sustainment strategy—Prepare for Sustainment—with Sustainment as Usual (SAU). Prepare for Sustainment (PFS) provides about half of the supervision support to implementers compared to what is typically provided during initial implementation. We examined the effects of these sustainment approaches on implementation fidelity and quality and student outcomes across two sustainability phases involving reduced support and no support.

### Conceptualizing sustainability as a process outcome

Sustainability refers to the ongoing delivery of an intervention with sustained health benefits and adherence to core program principles after initial training resources are withdrawn [18–20]. Scholars distinguish between sustainability as a process (e.g., planning for long-term implementation) and as an outcome (e.g., assessing how implementation strategies influence fidelity) [21]. The Integrated Sustainability Framework (ISF) [22] outlines multilevel factors that influence sustainment across settings, context, and populations [21–23] which informed our examination of sustainment-related implementation processes and outcomes. In this study, we assessed sustainability as the continued delivery of two group EBPs with adequate fidelity, penetration, and student benefit when implemented by school personnel.

### Multi-tiered systems of supports (MTSS) in schools

Many schools deliver mental health services within Multi-Tiered Systems of Support (MTSS), such as Positive Behavioral Interventions and Supports (PBIS) [24]. PBIS is a school-wide, multi-tiered framework grounded in a public health prevention model [25] that emphasizes teaching social behaviors that promote academic and social success [24]. The framework organizes supports across three tiers: universal prevention for all students (Tier 1), targeted group interventions for students with similar needs (Tier 2), and individualized interventions for students with significant concerns (Tier 3).

PBIS provides an effective structure for implementing targeted group evidence-based practices (EBPs) for students with or at risk for mental health difficulties [26]. Tier 2 services typically address shared externalizing concerns (e.g., disruptiveness, aggression) or internalizing concerns (e.g., anxiety, depression). Our research team has trained urban school personnel to deliver several Tier 2 EBPs [27–29], including the Coping Power Program for externalizing problems [30], group-based Primary and Secondary Control Enhancement Training (PASCET) for depressive symptoms [31, 32], and CBT for Anxiety Treatment in Schools (CATS) for anxiety symptoms [33, 34].

Group-based interventions allow schools to reach more students with fewer resources than individual therapy—an important advantage in under-resourced urban settings. All participating schools in this study had established Tier 1 systems and EBPs were implemented as Tier 2 supports for students with externalizing and internalizing difficulties.

### Training toward sustainability of EBPs in schools

Training for sustainability should equip implementers to maintain high fidelity to the EBP, given its strong association with child outcomes [35]. Yet fewer than half of EBPs— including those used in schools—are delivered with high fidelity during sustainment [7, 17, 22, 36]. Achieving fidelity in school settings is challenging because implementers, such as school counselors, often have limited formal training in EBPs before or after entering the workforce [37]. Providing training within the settings where implementers deliver services has been recommended to address these gaps, particularly in under-resourced communities [35, 38]. Evidence suggests that an initial training workshop paired with ongoing consultation is effective, whether delivered on-site or remotely [39]. However, additional research is needed to determine the optimal type and amount of training and consultation required to sustain high-fidelity implementation in under-served urban schools.

### Study aims

In this study, we examined sustainability of EBPs for externalizing behavior problems (Coping Power Program (CPP) [30] and anxiety problems (CBT for Anxiety Treatment in Schools; CATS) [33,34] for two implementation strategies: *Prepare for Sustainment* (PFS; a sustainment approach consisting of supervision provided by school district coaches to implementers supported by external consultants), compared to *Sustainment as Usual* (SAU; a sustainment as usual approach consisting of supervision provided by school district coaches without support from external consultants), on implementation and student outcomes [21]. Implementers in both conditions received the same training and consultation provided by research consultants during an initial implementation phase.

## Methods

### Study Design

The study used a two-arm randomized controlled trial with a Type 2 implementation-effectiveness hybrid design [40]. Schools were assigned to either PFS or SAU condition. The trial included three phases: *Phase 1-* Initial implementation; all EBP implementers received one year of direct training and consultation from a research consultant. Consultation occurred once for every EBP session delivered to students. Schools were randomized to conditions by the conclusion of Phase 1. *Phase 2* - Diminished Support (sustainment); implementers in PFS received year-long consultation from district coaches at a rate of one consultation for every two EBP sessions, while coaches themselves received support from the research consultant at the same rate. Implementers in SAU continued with their standard district supervision, and coaches did not receive consultation. *Phase 3* - No Support (sustainment); implementers in both conditions received supervision as usual for one year from district coaches, who no longer had research consultant support. Further methodological details are available in prior publications [41, 42].

### Specific aims and hypotheses

The study aimed to evaluate implementation outcomes—specifically intervention fidelity and service penetration—and student outcomes, including parent- and student-reported mental health symptoms and teacher-reported academic engagement, for PFS and SAU during Phases 2 and 3. We hypothesized that PFS would produce higher fidelity, greater penetration, and better student outcomes in Phase 2.

### Inclusion criteria

School district coaches were eligible if they were employed by the district, held a master’s degree, and were designated by district administrators to supervise implementers and serve as project champions.

School-based implementers were eligible if they had prior experience delivering group interventions, held a master’s degree, and were selected by the school principal to implement the study’s evidence-based practices.

Students were eligible if they attended a participating school, were enrolled in grades 4–8, were identified by teachers or implementers as requiring Tier 2 support, and scored more than one standard deviation above the mean on the Emotional Symptoms or Conduct Problems subscales of the Strengths and Difficulties Questionnaire (SDQ) [43], along with an Impact Supplement score of 1 (“a medium amount”) or 2 (“a great deal”) as rated by a parent or teacher.

### Exclusion criteria

School personnel not employed by the district and students not enrolled in Tier 2 services were excluded. Students with an “Intellectual Disability” classification or diagnoses that rendered participation clinically inappropriate (e.g., current substance use disorder, psychotic disorders, or autism spectrum disorders) were also excluded, as they were unlikely to benefit from the interventions or posed an acute safety risk.

### Participants

**Fig. 1:**
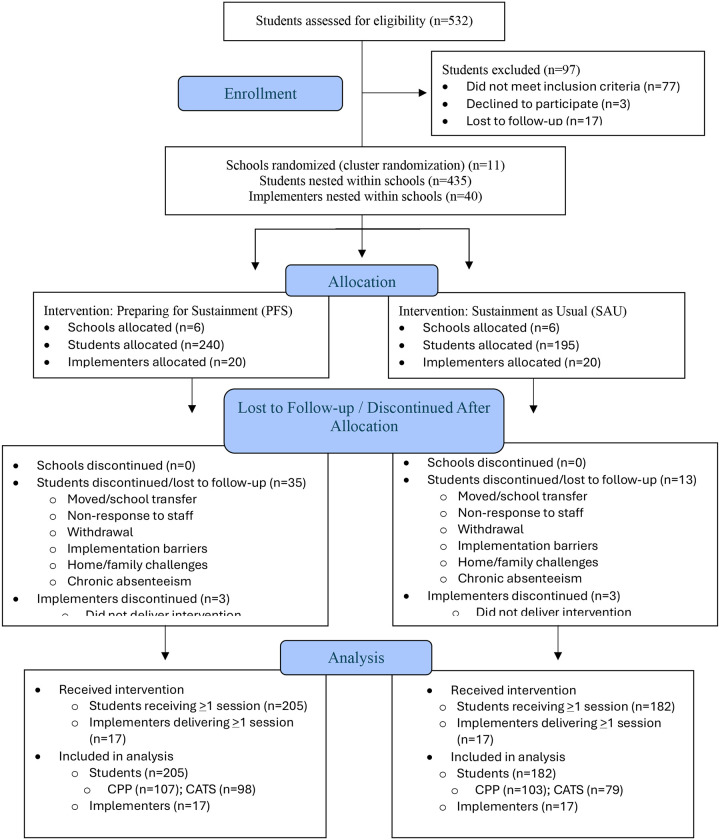
Consort flow diagram for the cluster-randomized study

A description of implementers and students who were eligible for the study, who consented and whose schools were randomly assigned to condition, implementers and students lost to follow up, and those included in final analyses is shown in CONSORT Figure 1.

### Implementers

Forty implementers (20 in PFS, 20 in SAU) were allocated to condition. Three implementers per condition discontinued participation, leaving 17 implementers per condition for analysis. Most implementers identified as female and African American and had a master’s degree level of education. One implementer had a doctorate degree in education. One participant did not report age or level of education. Thirty-three participants were school counselors, and one was a Social Emotional Learning teacher. There were no implementers self-identifying as Latino/Hispanic.

Twenty-three implementers participated in Phase 1, 23 in Phase 2 (12 in PFS; 11 in SAU), and 16 in Phase 3 (8 in PFS; 8 in SAU). Implementers did not differ on any of the demographic characteristics in any of the study phases (see Table 1).

### Students

A total of 532 students were screened for eligibility, and 435 enrolled in the study (PFS = 240; SAU = 195). Forty-eight students discontinued participation, yielding an analytic sample of 387 students (PFS = 205; SAU = 182). The sample included comparable numbers of male (PFS: 93; SAU: 94) and female (PFS: 110; SAU: 81) students. Most participants (n = 287) identified as African American. The two conditions differed significantly by race (χ^2^_(5)_ = 14.27, *p* = .014), ethnicity (χ^2^_(1)_ = 6.31, *p* = .012), and grade level (χ^2^_(4)_ = 11.85, *p* = .019), with SAU schools enrolling more African American and lower-grade students, and PFS schools enrolling more Hispanic/Latino students. No significant differences were observed for age, gender, primary residence, or baseline academic engagement or mental health symptoms. All participating schools were in low-income neighborhoods within a large Northeastern school district.

### Randomization

Participants were enrolled in three waves to optimize resource use. During Phase 1, all implementers received identical support from two research consultants to ensure consistent preparation for sustaining the interventions. At the end of each Phase 1 wave, schools were randomized 1:1 to either the PFS or SAU condition. Implementers, district coaches, and students followed the condition assigned to their school. Randomization was stratified by school level (elementary vs. middle) to maintain balance across conditions. One of the two consultants was assigned to support schools in the PFS condition during Phase 2.

### EBPs implemented by counselors

Implementers received training in the Coping Power Program (CPP) [30] for externalizing behavior problems and the CBT for Anxiety Treatment in Schools (CATS) [33, 34] program for anxiety. CPP comprises twelve 45-minute, CBT-based sessions and has demonstrated effectiveness in reducing aggressive behavior, covert delinquency, and substance use, with effects maintained at one-year follow-up [44]. Longitudinal analyses also indicate sustained reductions in aggressive and academic behavior problems for up to three years [45].

CATS is a manualized, group-based CBT program for anxiety, drawing on the Coping Cat [46] and FRIENDS for Life [47] protocols. The program teaches students to identify and manage physiological and cognitive symptoms of anxiety, develop coping plans, evaluate their progress, and use self-reinforcement. Evidence shows that CATS is equally effective but more cost-effective than FRIENDS [48, 49] (see Table 2).

Implementers assigned students to CPP if they met inclusion criteria on the SDQ plus a Total score > 12 on the Social Behavior subscale of the Social, Academic, and Emotional Behavior Risk Screener (SAEBRS) [50, 51]. Implementers assigned students to CATS if they met inclusion criteria on the SDQ plus a Total score of ≥ 25, or an elevated subscale score in one of four subscales: Panic > 7, Generalized Anxiety > 9, Separation Anxiety > 5, or Social Anxiety > 8 on the Screener for Child Anxiety Related Disorders (SCARED) [52] completed by a parent. CPP and CATS were implemented in group format during the lunch period.

### Theoretical frameworks

All training, consultation, and supervision activities for consultants, district coaches, and implementers were guided by the Interactive Systems Framework (ISF) [53]. The ISF comprises three components: the Synthesis and Translation System (STS), the Support System (SS), and the Delivery System (DS). The STS distills and prepares innovations for implementation; the SS provides the necessary supports for those delivering the interventions; and the DS carries out the interventions in real-world settings [53]. In this study, the STS was used to translate implementation and effectiveness data for CPP and CATS to participating schools and to engage principals in sustaining these EBPs. The SS coordinated support for consultants, coaches, and implementers, while the DS organized the delivery of the interventions by implementers [41].

The examination of sustainment outcomes was guided by the Integrated Sustainability Framework (ISF) [22]. We assessed how training and consultation influenced implementation fidelity and penetration, as well as student outcomes related to academic engagement and mental health.

### Prepare for sustainment (PFS) condition

Following Phase 1, schools were randomized in three waves to either PFS or SAU. Procedures for the PFS condition were conducted by the EBP implementers, a school district coach, and a research consultant. A licensed clinical psychologist supervised the consultant. After completing the Phase 2 training workshop, the district coach—supported by a research consultant—provided six remote consultation sessions for CPP implementers and four sessions for CATS implementers. Sessions were scheduled at the start of the school year. For each consultation session, implementers selected a CPP or CATS session for the coach to review and were expected to raise questions or implementation challenges (e.g., conducting role-plays or addressing barriers). Consultation was delivered via Zoom and supported by a shared online platform containing manuals, fidelity forms, and preparation instructions. In Phase 2, the supervising psychologist delivered one supervision session to the consultant for each consultation session the consultant provided to the coach. All planned supervision and consultation sessions by the psychologist, consultant and coach were implemented as expected.

**Figure 2:**
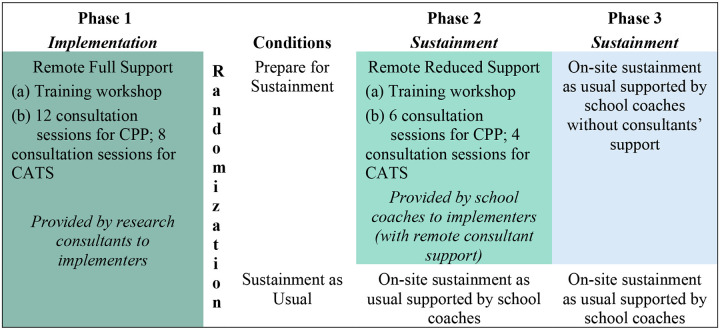
Sustainment support for school personnel

During Phase 3, the district coach continued supervising implementers in the PFS condition using a sustainment as usual approach. The coach no longer received support from the research consultant (see Figure 2).

### Sustainment as usual (SAU) condition

Procedures for the SAU condition were conducted by the implementers and a school district coach. During Phases 2–3 of the study, the school district coach provided separate remote supervision to the implementers using a sustainment as usual approach. The coach was instructed to provide supervision to implementers informed by the training the coach received during Phase 1. The coach was free to choose the frequency and length of the supervision sessions.

### Procedures

#### Training and consultation

The initial training workshop for coaches and implementers was delivered either on-site or remotely, as a single session or across multiple days based on scheduling needs. Ongoing supervision for implementers and consultation for district coaches were provided remotely via Zoom. Secure platforms—including REDCap, Box, and OneDrive—were used to share training materials, collect data, and facilitate access to recorded treatment and supervision sessions. Table 3 outlines the training procedures for coaches, implementers, and consultants across conditions.

#### Measures and data collection procedures

Data were collected on-site in schools, electronically through REDCap, or by phone. Four schools scheduled to implement interventions in Phase 3 did not provide data; therefore, Phase 3 analyses included only eight schools. All twelve schools contributed data during Phase 2. A full list of measures appears in Table 4.

### Data analytic plan

All analyses followed an intention-to-treat (ITT) framework. The study included three analytic phases. Phase 1, the pre-randomization period, was examined descriptively to characterize baseline conditions. Phase 2 served as the primary analytic phase for estimating causal effects of PFS versus SAU conditions. Phase 3, the follow-up period, was analyzed descriptively to evaluate maintenance of intervention effects.

Baseline demographic characteristics and student outcomes were compared across conditions to assess initial equivalence. Continuous variables were analyzed using independent two-sample *t*-tests or the Wilcoxon rank-sum test when normality assumptions were violated. Categorical variables were compared using Fisher’s exact test or χ^2^ tests, as appropriate. Two - tailed p-values are reported at the 0.05 alpha level.

The original analytic plan specified a mixed model for repeated measures (MMRM) with a hierarchical structure in which implementers were nested within schools and students were nested within implementers. The planned model included fixed effects for condition (PFS vs. SAU), time (baseline vs. post), and their interaction (condition × time). Random intercepts were specified at the school, implementer, and student levels to account for clustering and subject-level dependence over time. These predictors and random effects were used to estimate student outcomes. Analyses were to be conducted separately for CPP and CATS for student outcomes and reported for both Phase 2 and Phase 3.

As documented in [42], implementation barriers reduced the number of implementers, limiting the feasibility of multilevel modeling. Implementation fidelity was therefore analyzed using independent two-sample *t*-tests comparing pre- to post-intervention change scores between conditions, providing an indirect test of the condition × time interaction.

Implementation outcomes were examined using mean fidelity change scores (content and process fidelity) for CPP and CATS within each condition. Between-condition comparisons used two-sample *t*-tests or Wilcoxon rank-sum test. Between-condition comparisons used independent two-sample *t*-tests or Wilcoxon rank-sum tests. Service penetration was analyzed using the same approach.

Because randomization occurred at the school level (PFS vs. SAU) and implementers selected the treatment modality (CPP or CATS), analyses emphasized effect size estimation within modality and condition. Effect sizes were derived from MMRM models with students nested within implementers and implementers nested within schools. Outputs included least squares (LS) means and standard errors at baseline and post-intervention for each condition, Cohen’s *d* effect sizes, and 95% confidence intervals corresponding to the condition × time interaction.

Phase 2 data were used for all primary analyses addressing the main study aims. Phase 3 data were used for secondary analyses examining maintenance of effects. Phase 1 data were presented summarized descriptively because randomization occurred at its conclusion.

### Qualitative data

During the first wave of implementation, we conducted 52 qualitative interviews with 30 implementers, school administrators, coaches, and district administrators. Interviews were conducted prior to implementation, and again at the end of Phase 1, Phase 2, and Phase 3. Although there was some turnover among coaches and school staff, we re-interviewed the same individuals when staffing remained consistent. Interviews were digitally recorded, professionally transcribed, coded in NVivo, and analyzed thematically. Using an integrated approach [62] to codebook development, a priori codes were developed to capture relevant implementation constructs including acceptability and feasibility of training and sustainment supports and implementation and sustainment processes. Additional codes were added by the research team following a close reading of the first five transcripts [63]. Following coding, we conducted a team-based analytic review and member-checking process to compare interpretations and validate themes and conclusions.

## Results

### Phase 1:

There were no differences for content fidelity among implementers in schools that were randomized to PFS or SAU (*t*_[227]_ = .76; *Pr* > *|t|* = .458) for the implementation of CPP and CATS at the completion of Phase 1. Similarly, there were no differences between the conditions for process fidelity, including Active Engagement (Wilcoxon = 142.00; *Pr* > *|Z|* = .816) and Organized Teaching (Wilcoxon = 148.50; *Pr* > *|Z|* = .542). Also, there were no differences for service penetration between schools that were randomized to PFS or SAU (Fisher’s Two-sided Exact Test = .3506) at the completion of Phase 1.

Pre- to post-treatment academic engagement showed minimal change across all scales: Behavioral Engagement (17.22 [2.97] to 16.71 [2.24], mean = −0.51 [2.69]; 95% CI: −0.49 to 1.51); Behavioral Engagement (15.50 [3.78] to 15.51 [2.89], mean = −0.003 [3.43]; 95% CI: −1.28 to 1.27); Emotional Engagement (12.00 [2.79] to 12.20 [2.85], mean = −0.20 [2.86]; 95% CI: −1.25 to 0.84); Behavioral Disaffection (12.00 [2.79] to 12.20 [2.85], mean = −0.20 [2.82]; 95% CI: −1.25 to 0.84); and Emotional Disaffection (12.30 [3.89] to 12.80 [3.28], mean = −0.55 [3.65]; 95% CI: −1.91 to 0.81).

BASC-3 scores showed small reductions: The Externalizing score decreased from 65.5 (14.20) to 63.1 (13.97) (mean = 2.4 [14.10]; 95% CI: −2.8 to 5.5), and the Internalizing score decreased from 52.5 (10.43) to 51.1 (9.50) (mean = 1.3 [10.05]; 95% CI: −3.54 to 8.4).

BFS scores also changed minimally: My Conduct and Behavior declined from 10.29 (7.08) to 10.20 (6.80) (mean = 0.09 [6.96]; 95% CI: −2.50 to 2.68), and My Thoughts and Feelings declined from 8.09 (6.50) to 7.53 (7.00) (mean = 0.06 [6.71]; 95% CI: −1.93 to 3.05).

### Implementation Outcomes

#### Hypothesis 1

Implementers assigned to PFS will obtain higher content and process fidelity and service penetration (i.e., percentage of students receiving EBPs over total number of eligible students) at Phase 2 than implementers assigned to SAU, and those differences will be maintained during Phase 3.

##### Fidelity for Group EBPs.

**Phase 2:** As shown on Table 5, there were no differences between PFS and SAU for content fidelity (*t*_[21]_ = 1.41; *Pr* > *|t|* = .1725), or for Organized Teaching (process fidelity [Wilcoxon = 122.50; *Pr* > *|Z|* = .579]). However, there were differences between conditions for Active Engagement, where PFS (n = 12, mean = 3.150, [*SD* = .093]) had a higher process fidelity score than SAU (n = 11, mean = 3.009 [*SD* = .172]; Wilcoxon = 99.00; *Pr* > *|Z|* = .045).

**Phase 3**: There were no differences for content fidelity between PFS (n = 6, mean = 84.8, *SD* = 20.01), and SAU (n = 7, mean = 82.9, *SD* = 14.9]; *t*_[11]_ = .2; *Pr* > *|t|* = .842. Also, there were no group differences for Active Engagement (Wilcoxon = 49.00; *Pr* > *|Z|* = .350). However, implementers in PFS (n = 6, mean = 3.135, [*SD* = .118]) obtained a higher process fidelity score than SAU (n = 7, mean = 2.811 [*SD* = .201]) for Organized Teaching (Wilcoxon = 60.00; *Pr* > *|Z|* = .012).

#### Penetration

During Phase 2, 146 students were referred and 87 students received services across conditions (see Table 6). There were no differences between the two conditions among the number of students who were referred (n=80) and received services (n=46) in PFS schools, and the number of students who were referred (n=66) and received services (n=41) in SAU schools (Fisher’s Two-sided Exact Test = .7874). During Phase 3, 98 students were referred and 68 students received services across conditions. The penetration level was largely maintained during Phase 3, with no differences between PFS schools (52 students referred; 36 students served) and SAU schools (46 students referred; 32 students served; Fisher’s Two-sided Exact Test = 1.00).

#### Student Outcomes

##### Hypothesis 2

We expected that students receiving mental health interventions in PFS would show better academic engagement improvement and better symptom severity improvement at Phase 2 compared to their counterparts in SAU; and those differences would be maintained into Phase 3. Because we did not expect differences in academic engagement according to EBP, we combined for analysis the academic engagement data for both EBPs.

###### Academic Engagement with Learning

**Phase 2:** On the EvsD completed by teachers, there were medium effect sizes for Behavior Engagement (Cohen_*d* = .73), and Emotional Engagement (Cohen_*d* = .54), where students in PFS improved at post- compared to students in SAU (see Table 7).

**Phase 3:** Improvement was also observed for students in PFS compared to students in SAU. There was a large effect size for Emotional Engagement scale (Cohen_*d* = .85) and a medium effect for Behavioral Disaffection (Cohen_*d* = .71).

###### Externalizing Behavior Problems

Given that CPP was employed for students with externalizing problems and CATS was employed for students with anxiety (internalizing) problems, we report outcomes separately for students who received either EBP.

**Phase 2:** On the BFS-YR completed by students in the Coping Power Program (CPP) (Table 8), there was a large condition x time effect (Cohen_d = −.83) on the My conduct and Behavior scale; students in PFS self-rated as having a reduction in externalizing behavior problems at post- compared to students in SAU.

**Phase 3:** Across the two measures, the time-by-condition interaction was associated with small effect sizes (Cohen’s *d*).

###### Internalizing Problems

**Phase 2:** On the BFS-YR completed by students in the CBT for Anxiety Treatment in Schools (CATS), there was a medium size condition x time effect (Cohen_d = −.51) on the My Thoughts and Feelings scale; students in PFS self-rated as having a reduction in internalizing problems at post- compared to students in SAU.

**Phase 3:** Across the two measures, the time-by-condition interaction was associated with small effect sizes (Cohen’s *d*). See Table 8.

#### Perceptions of Training Support

##### Research Question 1

How will implementers perceive their training support?

**Staff appreciated external consultation**. Implementers appreciated the consultation they received. In the first year of support, interviewed implementers reported that consultation was “great” (IMP02 Y1) or “very supportive” (IMP07 Y1). Specifically, they noted benefits like helping them “cater the next session a little better” (IMP04 Y1), providing “positive feedback, negative feedback, that was helpful” (IMP10 Y1), and providing emotional support (IMP05 Y1). One of these implementers said that without the consultation, “I think I would have given up” (IMP05 Y1). Administrators also noted specific benefits to consultation, like ensuring that the school could “stay focused” on implementation (TTT15 Y1).

**Overall, those with reduced support in Year 2 would have liked continued external consultation**. While one implementer who joined a school in Year 2 and implemented for the first time without direct support from the consultant felt sufficiently supported by the training and district coaching (IMP28 Y2), several noted that they could have used more support. One noted that they could have used “a little more feedback” from the district coach in their second year implementing, but that would have been more of a challenge “if that was the situation for the first year” (IMP05 Y2). Similarly, an implementer (IMP06 Y2 and Y3) at the same school, felt that they would have benefited from consultation in Year 2 and Year 3, noting that the district coach had limited availability and time to support. Another thought their district coach did not have “enough knowledge” about the interventions to supervise effectively (IMP 32 Y2).

Those that desired continued consultation in Year 2 would have liked more support tailoring the intervention (IMP05 Y2) and “simple pointers” for dealing with group dynamics (IMP06 Y3). One implementer thought that it was more difficult for a new colleague to implement because the colleague had not received consultation in Year 1 (IMP05 Y3). Administrators reported that continued collaboration with the school district coach could have helped them better support the implementers (TTT12 Y2, TTT15 Y2). One district coach (C02 Y2) also reported wanting additional support, noting that they would have liked a consultant to support their first few supervisions.

## Discussion

The study aimed to compare implementation and student outcomes between a targeted sustainment strategy and sustainment as usual, and to examine implementers’ perceptions of training support in urban schools. The EBPs used in the study were implemented in the context of an existing PBIS (24, 25) program to support the teaching of students’ adaptive social behaviors via universal interventions. This work contributes to the evidence base on sustainment of mental health EBPs in under-resourced school settings, particularly regarding the role of external and internal training supports during sustainment [8, 22, 23].

The hypothesis that implementers in the PFS condition would demonstrate higher fidelity and greater service penetration than those in SAU was partially supported. PFS implementers consistently delivered EBP content with greater skill than implementers in SAU. In Phase 2, they engaged students more effectively during sessions; in Phase 3—despite the absence of study team support—implementers appeared warmer, more positive, and more organized in their delivery than their SAU counterparts. Content fidelity was high in both groups (89% in PFS vs. 83% in SAU) and the difference between groups was not statistically significant. Service penetration ranged from 58% to 70% across phases, with no significant between-group differences.

Qualitative data indicated that implementers who lacked prior support from trained school coaches wished they had received supervision on effective EBP delivery. Those who experienced reduced support in Phase 2 expressed a desire for more frequent supervision, noting personal and school-level benefits. School administrators similarly reported that continued collaboration with district coaches would have strengthened their ability to assist implementers.

Overall, the implementation findings indicate that reduced consultation for coaches and supervision for implementers after EBPs were established still enhanced implementers’ ability to engage students, and these skills were largely maintained even after consultation for coaches ended. This pattern is consistent with prior research highlighting the value of ongoing supervision or consultation following a period of initial implementation [14–16]. The results also suggest that providing roughly half of the original training intensity that is provided during the initial implementation of the EBPs is sufficient for preserving implementers’ clinical skills during sustainment. Although the combination of an initial workshop and reduced consultation for coaches improved implementer skill relative to training-as-usual, it did not increase content delivered or the number of students served. Notably, implementers in the training-as-usual condition still achieved acceptable levels of fidelity, skill, and service penetration, which is not consistent with findings from other studies [7, 36]. These findings suggest that under-resourced schools can feasibly sustain EBPs when implementers receive thorough initial training followed by supervision as usual.

Analysis of student outcomes showed several moderate and large treatment effects. For Phase 2, there were medium effect sizes for Behavior Engagement, and Emotional Engagement with learning, where students in PFS improved at post- compared to students in SAU. For Phase 3, there was a large effect size for the Emotional Engagement scale and a medium effect for the Behavioral Disaffection scale. The students in PFS appeared more emotionally engaged and motivated to learn, and less withdrawn and disengaged [61, 62] than students in SAU.

Regarding mental health outcomes, among students receiving CPP, those in PFS self-reported a large reduction in externalizing behavior in Phase 2. No moderate or large effects were observed in Phase 3 for students who received CPP. Among students receiving CATS in Phase 2, those in PFS reported moderate improvements in internalizing symptoms over those in SAU. No moderate or large effects were observed in Phase 3 for students who received CATS.

The student outcomes suggest that externalizing and internalizing symptomatology is likely to improve moderately in under-resourced schools when implementers use symptom-specific EBPs while receiving periodic supervision.

### Limitations

Due to a mismatch between the funding cycle and school calendar, implementation and student data for Phase 3 could not be collected in four schools. These schools were scheduled to implement the EBPs without research-team support, and because randomization occurred at the school level, this limitation is unlikely to have differentially affected conditions. Phase 2 results were not impacted.

Because students were not randomized to the treatment they received, some outcomes may have been influenced by pre-existing differences between students prior to receiving the treatment. This limitation is mitigated to some extent by prior evidence demonstrating that both EBPs used in the study are effective in under-resourced school settings. Even so, future research examining the comparative effectiveness of training strategies in schools would benefit from research designs that include random assignment of students to treatment.

Consistent with sustainability research in school settings, there was considerable staff turnover across study phases, preventing us from controlling for the exact training dosage each implementer received. Variability in training exposure may have influenced implementation quality and student outcomes.

Because the study was conducted in the regular school setting, implementers continued to receive supervision as usual from school administrators, and coaches continued to receive supervision as usual from district administrators. We were unable to account for potential additional mental health–related training or directives provided by administrators, which may have affected implementer performance. Future district-led studies may be better positioned to control for such contextual factors.

## Conclusion

This study shows a sustainment strategy consisting of an initial training for implementers and reduced consultation for school district coaches and supervision for implementers is effective for achieving high process fidelity and medium to large effects in academic engagement and clinical outcomes for students with externalizing and anxiety problems compared to a sustainment as usual strategy. Also, under-resourced schools might be able to sustain EBPs with acceptable fidelity when implementers receive thorough initial training followed by supervision as usual.

## Figures and Tables

**Table 1:** Demographic characteristics of implementers

Characteristic	Prepare for Sustainment PFS *n (%) / Mean (SD)*	Sustainment as Usual SAU *n (%) / Mean (SD)*	Statistical test	*P value*
EBP Implementers				
Gender (n=34)				
Male	3 (17.76)	4 (23.53)	Fisher Exact Test Pr	1.00
Female	14 (82.35)	13 (76.47)		
Age (n = 33)	44.06 (11.49)	47.18 (11.28)	t-test Pr	.44
Ethnicity (n = 34)*				
Not Hispanic	17 (100.00)	17 (100.00)		
Race (n = 34)				
White	6 (33.33)	7 (41.18)	Fisher Exact Test	1.00
Black/African American	11 (64.71)	10 (58.82)		

Note:

* None of the participants self-identified as Latino/Hispanic.

**Table 2:** Tier 2 interventions

Intervention	Description	Number of sessions	Implementer
Coping Power Program (CPP) [30]	Group intervention for children with, or at risk for, externalizing behavior problems	12	Counselor
CBT for Anxiety Treatment in Schools (CATS) [33,34]	Culturally-appropriate adaptation of Friends for Life (FRIENDS) [36] and *Coping Cat* [37] for children with, or at risk for, anxiety disorder	8	Counselor

**Table 3:** Training of coaches, implementers, and consultants across conditions

Coaches	EBP Implementers	Consultants
School district coaches in both PFS and SAU completed a 2-day training workshop led by research consultants on supervision strategies for implementing EBPs, including review of a supervisor competency framework (54), identifying eligible students, tracking progress, and advocating for principal and administrative support to ensure implementers had adequate time for preparation, supervision, and delivery. During Phase 1, coaches observed and assisted consultants twice as they conducted training and consultation with implementers. In Phases 2 and 3, coaches provided ongoing supervision to implementers and collaborated with administrators to boost administrator support for implementers.	School EBP implementers in both conditions completed an initial 8-hour training workshop delivered by research consultants. Training and consultation emphasized core adult learning principles (e.g., learning from experience, reflective practice, self-motivation) [12, 55] During Phase 1, consultants provided one consultation session for each group session delivered (12 for CPP; 8 for CATS). In Phases 2 and 3, implementers received supervision from district coaches. Consultation and supervision sessions included (a) reflecting on the prior group session, (b) discussing intervention strategies, and (c) problem-solving implementation barriers. Consultants and coaches also provided didactics, such as preparing for upcoming sessions and addressing questions about the consultation/supervision manual.	Consultants (two master’s-level clinicians) completed a three-step training process. First, they reviewed CPP and CATS treatment manuals and child workbooks to gain an overview of the protocols. Second, they attended a 3-hour workshop led by a licensed clinical psychologist with expertise in school-based EBPs, which included didactics, video examples, live modeling, and role-plays. Third, during Phase 1, the psychologist provided ten biweekly 60-minute supervision sessions, informed by review of recorded consultation sessions. Supervision emphasized accurate communication of EBP principles, reinforcement of supervisors, and consistent delivery of didactics. Consultants also participated in annual refresher trainings.

**Table 4:** Measures by variable/construct, measure characteristics, timepoint, method and informant

Variable/Construct	Measure	Measure characteristics	Timepoint	Method	Informant
Pre-Trial Activities					
Screening	Strengths and Difficulties Questionnaire (SDQ) [43] with Impact Supplement [56]	The SDQ is a 25-item, 3-point scale (0 = not true; 2 = certainly true) questionnaire used to assess the psychological adjustment of children and youth, ages 4–17.	Pre-treatment	Rating scale	Parents/teachers
Group assignment	Social, Academic, and Emotional Behavioral Risk Screener (SAEBRS) [50]	The SAEBRS is a 19-item screening instrument for emotional and behavioral risk. The SAEBRS is completed by school staff using a 0 (Never) to 3 (Almost always) Likert scale. It has excellent psychometric properties. It was used to assign students to CPP.	Ongoing	Rating scale	Teachers
	Screener for Child Anxiety Related Disorders (SCARED) [52]	The SCARED is a 41-item questionnaire using a 0 (Not True or Hardly Ever True) to 2 (Very True or Often True) scale used to screen for children with anxiety disorders, using five sub-scales and a Total Score. It has excellent psychometric properties and has been used in community settings as a screening instrument for anxiety disorders. It was used to assign students to CATS.	Pre-intervention	Rating scale	Parents
Compare sustainment conditions					
Implementation outcomes					
Content fidelity	CPP and CATS Content Fidelity Checklist (CFC) [29, 57]	The CFC reflects each activity component of the session agenda of CPP and CATS. Raters used a yes/no response scale to indicate whether the implementer covered a particular component of CPP and CATS as captured in audio recordings of the group sessions.	Ongoing	Coding	Research staff
Process fidelity	Process Fidelity Checklist (PFC) [29] for CPP and CATS	The PFC is a 10-item checklist rated on a scale of 1 to 5, with 1 being *Not at all* and 5 being *Very Often*. Ratings were given on the extent to which implementers actively engaged students in session and delivered the intervention in an orderly fashion, using active learning strategies and examples that are relevant to the students	Weekly	Coding	Research staff
Penetration	Penetration Inventory (PI)	The PI is an Excel track sheet listing referrals issued by teachers for intervention in grades 4–8 and students who received one of the two EBPs.	Monthly	Quan	Implementers/District coaches
Student outcomes					
Mental health symptoms	Behavior Assessment System for Children- 3^rd^ Edition (BASC-3) [58]	The BASC-3 is a 138-item, 4-point (1=Never, 2=Sometimes, 3=Often, 4=Almost always) rating scale for assessing parental report of child mental health functioning, standardized for ages 2.5 to 18 years. The BASC-3 has excellent psychometric properties. The Externalizing and Internalizing scales were used to measure symptom change.	Pre/Post-treatment	Rating scale	Parent
	The Behavior and Feeling Survey-Youth Report (BFS-YR) [59]	The YFS-SR is a 10-items measure of internalizing (My Thoughts and Feelings) and externalizing (My Conduct and Behavior) problems rated on a 5-point scale (0 = not a problem; 4 = a very big problem).	Pre/Post-treatment	Rating scale	Student
Student academic engagement	Engagement versus Disaffection with Learning- Teacher Report (EvsD-Teacher) [60, 61]	The EvsD Teacher is a 20-item, four-point (1 = not at all true; 4 = very true) instrument with four sub-scales: Behavioral Engagement, Emotional Engagement, Behavioral Disaffection and Emotional Disaffection. We used the average score for each of the four scales.	Pre/Post-treatment	Rating scale	Teacher

**Table 5:** Content and process fidelity by EBP and condition

	Prepare for Sustainment		Sustainment as Usual	
	PFS		SAU	
	*Mean SD*		*Mean SD*	
Phase 2				
Content Fidelity	88.5%	.10	82.82%	.10
Active Engagement*	3.15	.09	3.01	.17
Organized Teaching	3.03	.16	2.98	.21
Phase 3				
Content Fidelity	84.83%	.20	82.86%	.15
Active Engagement	3.09	.11	3.00	.15
Organized Teaching**	3.14	.12	2.81	.20

Note:

* PFS VS. SAU *Pr *> |*t*| = .029;

** *Pr *> |*t*| = .005

**Table 6:** Service penetration by condition and study phase

	Prepare for Sustainment			Sustainment as Usual		
	Referred *N (%)*	Served *N (%)*	Penetration *%*	Referred *N (%)*	Served *N (%)*	Penetration *%*
Phase 2	80 (63.49)	46 (36.51)	57.5	66 (61.68)	41 (38.32)	62.1
Phase 3	52 (59.09)	36 (40.91)	69.2	46 (58.97)	32 (41.03)	69.6

**Table 7:** Effect sizes (Cohen’s d) and 95% confidence intervals for Time × Condition interactions, indicating the magnitude of differential change over time between conditions across engagement and disaffection with learning outcomes.

Outcome Measure	PFS condition		SAU Condition		Df	Cohen_d	d_95%CI	Effect Size_d rank
	Pre *M(SE)*	Post *M(SE)*	Pre *M(SE)*	Post *M(SE)*				
**Phase 2**								
Teacher report	(n = 76)		(n = 63)					
EvsD*								
Behavioral								
Engagement	10.96 (0.58)	12.03 (0.59)	11.04 (0.62)	11.75 (0.63)	115	0.73	−.05, 1.41	Medium
Emotional								
Engagement	11.52 (0.54)	13.05 (0.56)	12.26 (0.57)	12.82 (0.60)	115	0.54	−.14, 1.22	Medium
Behavioral								
Disaffection	11.57 (0.53)	12.24 (0.55)	11.49 (0.58)	12.32 (0.59)	115	0.26	−0.41, 0.94	Small
Emotional								
Disaffection	12.36 (0.47)	13.52 (0.49)	12.98 (0.51)	14.35 (0.52)	115	0.27	−0.40, 0.94	Small
**Phase 3**								
Teacher report	(n = 48		(n = 40)					
EvsD (n=59)								
Behavioral								
Engagement	10.52 (0.63)	12.18 (0.67)	10.89 (0.86)	11.54 (0.78)	59	0.85	−0.08, 1.79	Large
Emotional								
Engagement	11.83 (0.59)	13.36 (0.65)	12.78 (0.84)	12.90 (0.74)	59	0.26	−0.66, 1.19	Small
Behavioral								
Disaffection	11.14 (0.51)	12.74 (0.57)	11.36 (0.51)	11.13 (0.65)	59	0.71	−0.20, 1.63	Medium
Emotional								
Disaffection	11.95 (0.53)	13.58 (0.59)	12.98 (0.76)	13.18 (0.66)	59	0.46	−0.44, 1.37	Small

* (EvsD) Engagement versus Disaffection with Learning.

**Table 8:** Mixed-model repeated measures results for the Coping Power Program and CBT Treatment for Children in Schools, including parent and student rating scales across Phase 2 and Phase 3.

Outcome Measure	PFS condition		SAU Condition		Df	Cohen_d*	d_95%CI*	Effect Size_d rank
	Pre *M(SE)*	Post *M(SE)*	Pre *M(SE)*	Post *M(SE)*				
**Coping Power Program**								
**Phase 2**								
Parent Report								
MASC 3** (n=75)								
Externalizing Problems	65.381 (3.11)	62.624 (3.20)	63.646 (3.11)	62.427 (3.11)	47	−.26	−1.04, .54	Small
Student Report								
BFS-YR** (n=81)								
My Conduct and Behavior	8.512 (1.48)	5.567 (1.57)	8.728 (1.34)	9.009 (1.41)	51	−.83	−1.57, −.08	Large
Parent Report								
MASC 3 (n=52)								
**Phase 3**								
Parent Report								
MASC 3 (n=52)								
Externalizing Problems	71.135 (2.52)	63.319 (2.88)	60.187 (2.28)	55.644 (2.43)	30	−.47	−1.38, .46	Small
Student Report								
BFS-YR (n=59)								
My Conduct and Behavior	12.040 (1.12)	8.104 (1.25)	9.770 (.95)	7.520 (1.10)	40	−.42	−1.27, .43	Small
**CBT Anxiety Treatment in Schools**								
**Phase 2**								
MASC 3** (n=61)								
Internalizing Problems	56.237 (1.90)	56.230 (1.99)	56.442 (2.28)	53.964 (2.49)	37	−.33	−.50, 1.45	Small
BFS-YR** (n=64)								
My Thoughts and Feelings	12.930 (1.00)	11.107 (1.03)	7.251 (1.24)	7.287 (1.32)	48	−.51	−1.31, .28	Medium
**Phase 3**								
MASC 3* (n=39)								
Internalizing Problems	52.313 (2.90)	51.195 (2.91)	56.493 (3.52)	55.045 (3.57)	37	.09	−1.12, 1.30	Small
BFS-YR* (n=40)								
My Thoughts and Feelings	10.857 (1.27)	7.461 (1.29)	13.606 (1.93)	11.303 (2.20)	33	−.2	−1.29, .88	Small

* Cohen’s d and the associated 95% confidence intervals reflect effect sizes derived from the time × condition interaction terms from the MMRM models.

** (BFS-YR) Behavior and Feeling Survey-Youth Report; (BASC 3) Behavior Assessment System for Children – Third Edition.

## Data Availability

De-identified data used in the study can be obtained upon reasonable request from the first author.

## Abbreviations

BASC 3: Behavior Assessment System for Children- 3^rd^ Edition
BFS-YR: The Behavior and Feeling Survey-Youth Report
CATS: CBT for Anxiety Treatment in Schools
CFC: Content Fidelity Checklist
CPP: Coping Power Program
EBP: Evidence-based practice
EvsD: Engagement versus Disaffection with Learning
FRIENDS: Friends for Life
ISF: Interactive System Framework
ISF: Integrated Sustainability Framework
ITT: Intention-to-treat
LS: Least squares
MMRM: Mixed model for repeated measures
MTSS: Multi-tiered Systems of Support
PBIS: Positive Behavioral Interventions and Supports
PI: Penetration Inventory
PFC: Process Fidelity Checklist
PFS: Prepare for Sustainment
REDCap: Research Electronic Data Capture
SAEBRS: Social, Academic, and Emotional Behavior Risk Screener
SAU: Sustainment as Usual
SD: Standard deviation
SE: Standard error
SDQ: Strengths and Difficulties Questionnaire
SCARED: Screener for Child Anxiety Related Disorders
